# Building on the clinical applicability of ctDNA analysis in non-metastatic pancreatic ductal adenocarcinoma

**DOI:** 10.1038/s41598-024-67235-y

**Published:** 2024-07-13

**Authors:** Ibone Labiano, Ana E. Huerta, Maria Alsina, Hugo Arasanz, Natalia Castro, Saioa Mendaza, Arturo Lecumberri, Iranzu Gonzalez-Borja, David Guerrero-Setas, Ana Patiño-Garcia, Gorka Alkorta-Aranburu, Irene Hernández-Garcia, Virginia Arrazubi, Elena Mata, David Gomez, Antonio Viudez, Ruth Vera

**Affiliations:** 1 Oncobiona Group, Navarrabiomed-Instituto de Investigación Sanitaria de Navarra (IdiSNA), Irunlarrea 3, 31008 Pamplona, Spain; 2 Medical Oncology Department, Hospital Universitario de Navarra (HUN), Irunlarrea 3, 31008 Pamplona, Spain; 3 Molecular Pathology of Cancer Group, Navarrabiomed, Hospital Universitario de Navarra (HUN), Instituto de Investigación Sanitaria de Navarra (IdiSNA), Irunlarrea 3, 31008 Pamplona, Spain; 4 Department of Pediatrics and Clinical Genetics, Clínica Universidad de Navarra (CUN), Cancer Center Clínica Universidad de Navarra (CCUN), Program in Solid Tumors, Center for Applied Medical Research (CIMA) and Navarra Institute for Health Research (IdiSNA), University of Navarra, Pamplona, Spain; 5 CIMA LAB Diagnostics, University of Navarra, Pamplona, Spain

**Keywords:** Liquid biopsy, Genomics, Biomarkers, Precision medicine, Gastrointestinal neoplasms, Gastrointestinal cancer, Tumour biomarkers, Cancer, Biomarkers, Oncology, Genetics, Genetic markers

## Abstract

Pancreatic ductal adenocarcinoma represents one of the solid tumors showing the worst prognosis worldwide, with a high recurrence rate after adjuvant or neoadjuvant therapy. Circulating tumor DNA analysis raised as a promising non-invasive tool to characterize tumor genomics and to assess treatment response. In this study, surgical tumor tissue and sequential blood samples were analyzed by next-generation sequencing and were correlated with clinical and pathological characteristics. Thirty resectable/borderline pancreatic ductal adenocarcinoma patients treated at the Hospital Universitario de Navarra were included. Circulating tumoral DNA sequencing identified pathogenic variants in *KRAS* and *TP53,* and in other cancer-associated genes. Pathogenic variants at diagnosis were detected in patients with a poorer outcome, and were correlated with response to neoadjuvant therapy in borderline pancreatic ductal adneocarcinoma patients. Higher variant allele frequency at diagnosis was associated with worse prognosis, and thesum of variant allele frequency was greater in samples at progression. Our results build on the potential value of circulating tumor DNA for non-metastatic pancreatic ductal adenocarcinoma patients, by complementing tissue genetic information and as a non-invasive tool for treatment decision. Confirmatory studies are needed to corroborate these findings.

## Introduction

Pancreatic ductal adenocarcinoma (PDAC) represents a worldwide problem. With an all stages 5 year overall survival of 9%^1,2^, PDAC ranks the seventh-leading cause of cancer-related death^3^. Furthermore, its incidence has been increasing during the last 20 years and it has been projected to become the third leading cause of cancer-related death by 2025^4^. Curative options for PDAC patients are scarce and, despite research efforts, few advances have been achieved in the last years.

Surgery is the only curative treatment option and resectability of PDAC patients relies on the exclusion of vascular tumor involvement, in order to provide an R0 resection^1^. Subsequently, adjuvant chemotherapy is indicated, with an intention to treat possible persistent tumor cells and improve the survival outcomes of these patients^5,6^. In patients presenting borderline PDAC (defined by limited vascular involvement technically resectable or elevated baseline levels of the carbohydrate antigen (CA) 19-9)^7,8^, induction chemotherapy and optionally chemoradiotherapy treatment are recommended, with the aim of tumor down staging and potentially eradicating micrometastatic disease, to assure a secondary curative surgery^1^. Although the type of neoadjuvant chemotherapy is still a matter of debate^9,10^, some genetic alterations may guide treatment decisions, such as the preference of platinum combinations in patients with mutations in the homologous recombination genes^11^.

Unfortunately, even after tumor resection, the vast majority of patients will eventually present recurrence or disease progression. In this scenario, non-invasive biomarkers that provide real-time information on tumor biology represent a valuable tool to monitor tumor evolution and improve patient follow-up. In line with this, the potential information derived from the analysis of circulating tumor DNA (ctDNA) raises as a promising non-invasive tool to monitor disease evolution while obtaining real-time genetic information of the tumor during treatment^12,13^.

The usefulness of ctDNA is mostly stablished in lung, breast, prostate and colon cancer, where clinical trials have assessed its potential value for early diagnosis, detection of minimal residual disease, selection of patients for targeted therapies and monitoring of tumor evolution^14^. In the case of PDAC, several studies have revealed the valuable role of ctDNA analysis in different time-points along tumor evolution^15^. In this regard, plasma ctDNA may serve as an early-diagnostic marker, ctDNA amounts increase in patients with more advanced disease and hold a prognostic value^16–18^. In the setting of resectable PDAC, genetic characterization on the tumor tissue biopsy remains the gold standard, and studies report varying results when assessing concordance of NGS analyses on ctDNA and tumor tissue^19,20^. Additionally, ctDNA arises as a promising tool to monitor minimal residual disease after surgery^21,22^ and also response to treatment^23^. Nevertheless, real clinical application of ctDNA for PDAC is still far from clear and needs further validation^13,24^.

In the current manuscript, we present the results of a prospective ctDNA analysis in a cohort of patients with resectable and borderline PDAC diagnosed and treated in the Hospital Universitario de Navarra (HUN), Spain. This study is part of two consecutive research projects for the development of precision medicine in cancer, granted by the Government of Navarra (DIANA (ref: 0011-1411-2017-000033) and AGATA (ref: 0011-1411-2020-000013)).

## Materials and methods

### Patient recruitment and study design

This study included 30 patients diagnosed with resectable and borderline PDAC, recruited at the Medical Oncology Department of the HUN between November 2017 and August 2020. Epidemiological and clinical variables, including radiological assessment, CA 19-9 levels and treatment procedures were recorded. Written informed consent was obtained for all patients. The study was approved by the Clinical Research Ethics Committee of the HUN, in line with the ethical guidelines of the 1975 Declaration of Helsinki (sixth revision, 2008) as reflected in a priori approval by the institution’s human research committee (PI_2020-118).

### Next-generation sequencing (NGS) of plasma samples

Blood samples were collected by trained nurses of the Medical Oncology Department of HUN, at different time points during disease evolution: at diagnosis (baseline plasma), at the time of the first tumor radiological assessment after neoadjuvant chemotherapy (first tumor assessment plasma), and at the time of diagnosis of the clinical progression (progression plasma). In each time point, 10 mL of blood were collected in vacutainer-EDTA tubes, samples were processed for plasma separation by centrifugation within 2 h after extraction, and stored at −80 ℃ in the Biobank of Navarrabiomed until use. Circulating total nucleic acids (TNA) were extracted from plasma using MagMAX™ Cell-Free Total Nucleic Acid Isolation Kit (ThermoFisher) and concentration and integrity of nucleic acids were checked with Qubit (ThermoFisher) and TapeStation (Agilent). Libraries were prepared for amplicon enrichment and barcode attachment with the Oncomine™ Pan-cancer Cell-Free Assay kit (ThermoFisher) in an Ion Chef™ (ThermoFisher) and sequenced in a S5 sequencer with 540 chips following manufacturer recommendations. The Oncomine™ Pan-cancer Cell-Free Assay enables the identification of genetic alterations including copy number variations (CNVs), RNA exon variants, fusions and single or multiple nucleotide variants (SNVs or MNVs, respectively) as well as short insertions and deletion (indels) in 52 genes related to cancer biology.

### NGS of tissue-surgery samples

Tissue sections were obtained by the microdissection of histologically-confirmed tumor tissue of FFPE specimens derived from surgical resection by pancreatic-duodenectomy (Whipple procedure). A total of 8–10 tissue sections of 5 μm were employed for circulating TNA extraction in a Maxwell automated system (Promega), concentration and integrity were checked with Qubit (ThermoFisher) and TapeStation (Agilent). Libraries were prepared for amplicon enrichment with the Oncomine™ Comprehensive v3 Assay kit (ThermoFisher) in an Ion Chef™ (ThermoFisher) and sequenced in a S5 sequencer with 540 chips following manufacturer’s recommendations. The Oncomine™ Comprehensive v3 Assay enables the identification of CNVs, RNA exon variants, fusions and DNA variants including SNVs or MNVs and indels in 161 genes related to cancer biology.

### Sequencing data analyses and variant calling

Torrent Suite Software version 5.16.1 was used to check quality criteria metrics of each sequencing run following manufacturer recommendations. Raw reads were aligned to the human reference genome GRCh37-hg19 and variant calling was performed on the Ion Reporter software v.5.10. The Oncomine TagSeq Pan-Cancer Liquid Biopsy w2.1—Single Sample r.0 workflow and the Oncomine Pan-Cancer Assay Baseline v1.0 copy number baseline were employed for liquid plasma ctDNA analyses. For tissue analyses, the Oncomine Comprehensive v3-w3.2 -DNA and Fusions—Single Sample r.0 workflow and the Oncomine Comprehensive DNA v3 540 Assay Baseline v2.1 copy number baseline were employed.

Variant filtering was performed on the non-filtered-oncomine.tsv files with a customized filtering pipeline. CNVs were assessed when median absolute pairwise difference (MAPD) QC was passed and accepted if the number of copies at the lower 5% of the CI was ≥ 4. RNA exon variants and fusions were assessed only if control gene QCs were passed (*TBP*, *HMBS* genes for plasma ctDNA Pan-Cancer and *TBP*, *MRPL13*, *MYC*, *HMBS*, *ITGB7* and*LRP1* genes for tissue/Comprehensive v3). Exon 14 skipping in *MET* (METex14) was only assessed when *MET* wild-type QC was passed and accepted when > 50 reads representing the exon skipping were identified. Filter chain was: coverage > 500x, allele reads > 2 (only for plasma samples), allele frequency > 0.01% for plasma samples and 5–50% for tissue samples (except for two variants in *KRAS* that were accepted with 4.02 and 4.37% of frequency, based on the extensively reported role of these variants in PDAC), not present in 500 Exomes and Named Variants, location = exonic/splicing variant (variants located in intronic and UTR filtered out), function = missense / unknown (variants leading to synonymous alterations filtered out). An additional filter was employed for *TP53* variants, variants were not included if they were not present in the TP53 variant database from the National Cancer Institute. Variants were classified according to their pathogenicity based on American College of Medical Genetics criteria^25^ using the Varsome database, as “Benign”, “Likely benign”, “Variants of Unknown Significance (VUS)”, “Likely Pathogenic” or “Pathogenic. “Benign” and “Likely benign” were filtered out. Copy number gains, fusions, and gene rearrangements were considered pathogenic.

Additionally, in order to gain insight into the origin of the genetic variants detected in ctDNA two complementary approaches were employed. First, each variant was searched in the Catalogue of Somatic Mutations in Cancer (COSMIC) database, recording both the number of patients with pancreatic cancer in which each alteration was found and other malignancies where the same alteration was detected. Next, given than clonal hematopoiesis of indeterminate potential (CHIP) is the most common source of confounding variants in cfDNA, a comparison was made with variants from a recently publishing study investigating CHIP-related variants over 50,000 individuals, with the database of identified variants publicly available^26^.

### Statistical analysis

R studio v.4.2.1. and GraphPad Prism v. 9.0 software platforms were used for statistical analyses. Quantitative variables are reported with median, q1 and q3, while qualitative variables are reported with n and percentages. For comparisons, patients were divided according to the presence or lack of pathogenic alterations. Patients presenting no genetic alterations or only presenting VUS, or likely pathogenic variants were included in the “non-pathogenic” group, while patients presenting at least one pathogenic variant comprised the “pathogenic” group. In addition, borderline PDAC patients initially treated with neoadyuvant therapy (NAT) were divided according to the tumor radiological treatment response as disease control “yes” (i.e. the sum of complete/partial response and stable tumors) and “no” for progression tumors. The response was evaluated after NAT on tumor size and vascular invasion. Progression was defined with the following criteria: increase of tumor size, increase of vascular invasion and presence of new malignant images. Disease control was defined with the contrary criteria. Non-parametric Mann-Whitney U test was employed for comparison of quantitative variables and Fisher’s exact test for qualitative variables. Cramer`s V test was used to evaluate the effect size of association, considering as moderate, values between 0.2 and ≤ 0.6, and as strong values > 0.6. Overall survival (OS) and event-free survival (EFS) were evaluated by the Kaplan-Meier method and the log-rank test was used to assess differences in survival time between groups. Hazard ratio (HR) was obtained with Cox regression model. Survival time was defined as the period of time in months from the date of diagnosis to the date of death. A p-value less than 0.05 was considered as statistically significant.

## Results

### Patients characteristics

**Figure 1:**
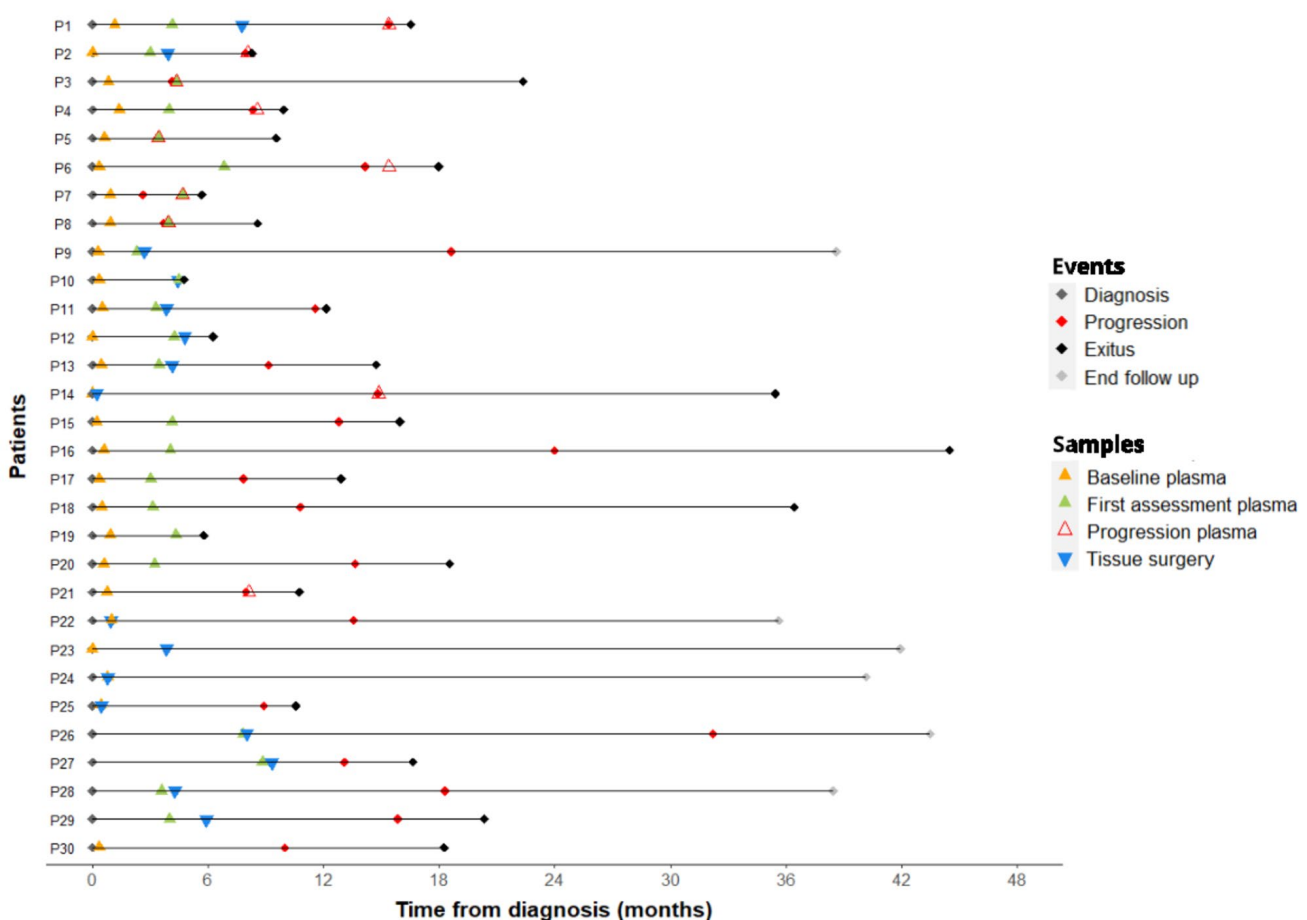
Clinical events and sample collection during follow-up. Horizontal lines represent the time frame from diagnosis to the end of the follow-up for each patient. Colored diamonds represent clinical events including diagnosis (grey), progression (red), exitus (black) and end of follow-up (grey). Colored triangles represent samples collected from each patient, tissue-surgery (blue) and plasma samples at different time points including, baseline plasma (orange), first tumor assessment plasma (green) and progression plasma (red).

The study included 30 patients with previous histological confirmation of PDAC. Patients´ characteristics are summarized in Table 1. Sixteen (53.3%) were female, with a median age of 66.7 years. According to previously mentioned criteria (vascular involvement and possibility of an R0 resection), at diagnosis, four patients (13%) were regarded as resectable and directly derived for primary surgery, while most of them (26 patients, 87%) were classified as border line and treated with a NAT. NAT was based in abraxane plus gemcitabine (n = 19; 73.1%) or FOLFIRINOX (n = 7; 26.9%). Patients received a median of 3 cycles (range: 3 to 12). Those patients whose tumors did not met response criteria enough for getting an adequate surgical resection, proceeded to additional 3 cycles of the same chemotherapy schema (n = 4; 44,4%) or chemoradiotherapy (n = 5; 55,6%) previous to the final surgery. Of the 26 patients treated with NAT, 16 (62%) showed disease control after treatment and could be forwarded to resection, while 10 (38%) showed progression of disease, as described by previously mentioned criteria (increase of tumor size, vascular invasion or presence of new malignancies). After inclusion, patients were followed in routine clinical practice; last follow-up update was in May 2023. Median follow-up was 16.6 months. Figure 1 shows follow up, clinical course and samples collected for each patient. Overall, 59 plasma samples were obtained at different time-points: 26 at diagnosis before any onco-specific treatment, including surgery (baseline plasma), 23 at the first tumor radiological assessment after NAT (first tumor assessment plasma), and 10 at the time of tumor progression (progression plasma). Tissue samples from surgery were obtained from 16 patients.

**Table 1:** Clinico-pathological parameters of the patients.

Characteristics		Patients n = 30
Age (years)*		66.7 (57.5–75.2)
Sex (n, %)	Male	14 (46.7)
	Female	16 (53.3)
Diagnostic clinical classification (n, %)	Resectable	4 (13.3)
	Borderline	26 (86.7)
	Disease control-yes	16 (61.5)
	Disease control-no	10 (38.5)
cT (n, %)	T1	2 (6.7)
	T2	14 (46.7)
	T3	9 (30)
	T4	5 (16.7)
cN (n, %)	N0	18 (60)
	N +	12 (40)
CA 19–9 (U/ml)*		350.5 (105.8–2,203.8)

*CA* carbohydrate antigen, *NAT* neoadyuvant treatment.

*Quantitative variables are described as median (Q1-Q3).

### Description of genetic alterations

**Figure 2:**
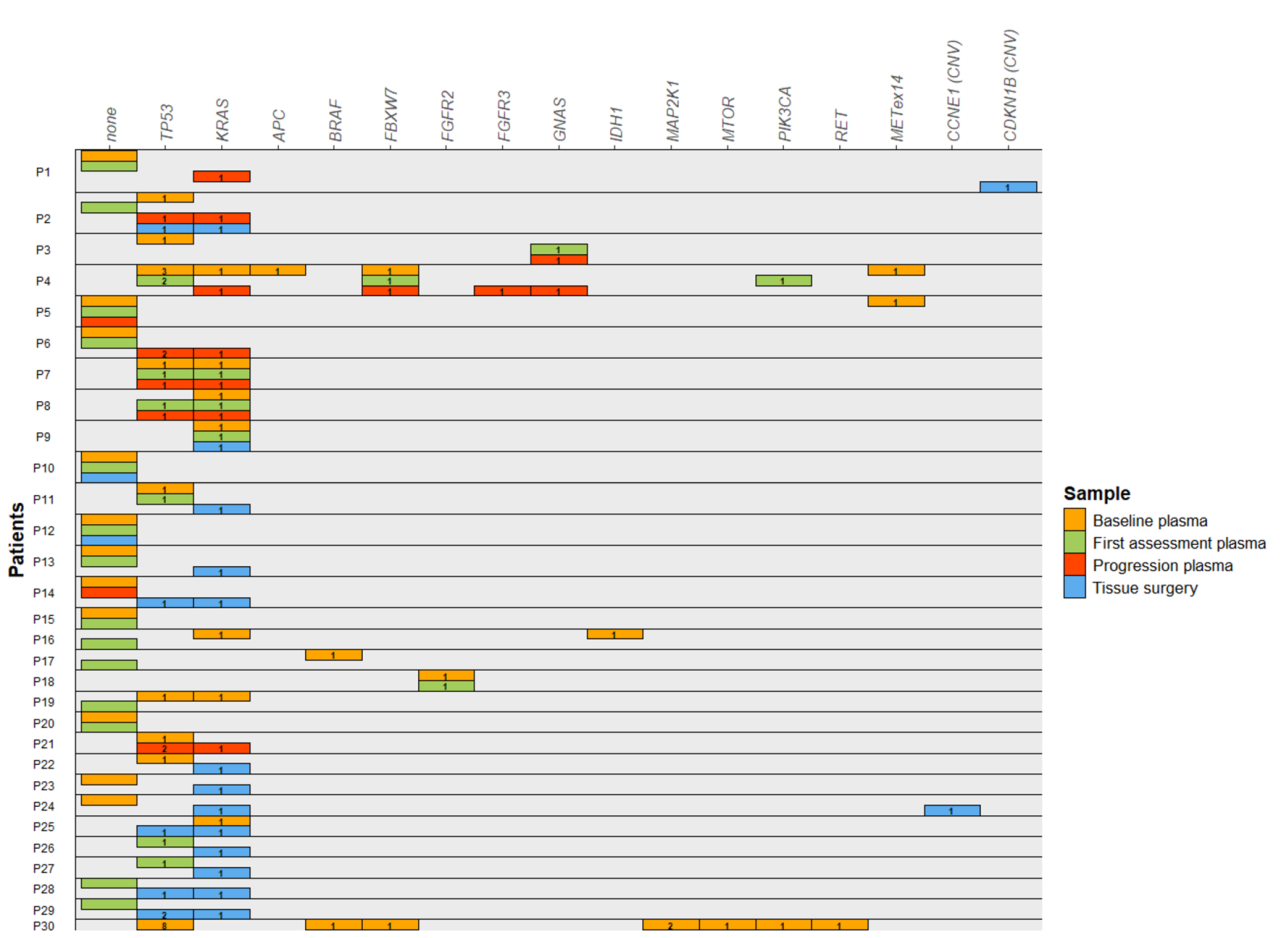
Summary of the pathogenic genomic alterations found in plasma and tissue samples from all patients. Horizontal boxes represent one patient, with one sub-line for each of the samples available for each patient. Colored rectangles represent the gene in which a genetic alteration was identified in each sample, including baseline plasma (orange), first tumor assessment plasma (green), progression plasma (red) and tissue surgery (blue). The label in each rectangle depicts the number of alterations observed for each gene.

Altered genes identified in plasma and tissue for each patient are summarized in Fig. 2. Specific genetic alterations identified in each plasma sample and in tissue for each patient are described in detail in Tables S1–S4, Fig. 1S depicts the percentage of pathogenic alterations detected in each gene by sample.

At diagnosis, sixteen patients (61.5%) presented at least one pathogenic alteration in baseline plasma samples, while 10 (38.5%) did not present any pathogenic alteration. The most frequently mutated genes were *TP53* (n = 9, 34.5%) and *KRAS* (n = 7, 26.9%). Other pathogenic variants in tumor suppressor genes (*APC, FBXW7*) and oncogenes (*FGFR2 and PIK3CA*) were also detected. Interestingly, besides genetic variants, *MET* exon 14 skipping was detected in two patients (Fig. 2, Table S1). Overall, most patients presented 1 or 2 pathogenic genetic alterations. It is worth mentioning that one patient (P30) presented up to 15 pathogenic variants. Unfortunately, we were not able to study P30 further due to the bad course of the disease.

Plasma samples after NAT treatment (first tumor assessment plasma) presented less genetic alterations. Only 9 patients (39.1%) showed pathogenic alterations. Similarly to what we observed in baseline plasma samples, the most frequently mutated genes were *TP53* (n = 5, 21.7%) and *KRAS* (n = 3, 13.0%). Again, other pathogenic variants were identified in different genes including *FBXW7*, *PIK3CA* and *FGFR2* (Fig. 2, Table S2).

Finally, plasma samples at tumor progression (progression plasma) were available for 10 patients. In this case, pathogenic alteration were detected in most of them (n = 8, 80%). Again, a similar landscape of altered genes was identified, being *KRAS* (n = 7, 70%) and *TP53* (n = 5, 50%) the most commonly mutated genes (Fig. 2, Table S3). Of note, certain genetic variants were only identified at progression, including *GNAS* (c.601C > T) and *FGFR3* (c.1948A > G).

Although the tumoral origin of genetic variants detected in ctDNA cannot be unequivocally proven, a search for each variant identified in plasma was conducted in the COSMIC database. This search revealed that, although few variants have been reported in studies focused in PDAC, most of the variants have already been reported in other solid and hematologic tumors. Additionally, when comparing the variants with those from a publicly available CHIP study, it was observed that some of the genes with identified variants were not referred to as common CHIP genes and thus were not included in the mentioned manuscript (i.e., *APC*, *FGFR2*, *PIK3CA*, *ESR1*, *AR*, *PDGFRA*, *FGFR3*, *MET*). Among the genes with detected variants that were also analyzed in this manuscript, only *KRAS* c.35G > T; p.Gly12Val was identified as associated to M-CHIP, which is also a well-known oncogenic variant in PDAC and other solid malignancies (Table S1–S3).

When analyzing tissue samples, pathogenic alterations were detected in 14 patients (87.5%). Again, *KRAS* (n = 13, 81.3%) and *TP53* (n = 5, 31.2%) were the most commonly mutated genes. Besides *KRAS* and *TP53*, no other pathogenic variants were identified. Interestingly, other genetic alterations including copy number gains in *CDKN1B* and *CCNE1* were identified in two different patients (Fig. 2, Table S4).

### Comparisons between baseline plasma ctDNA and tissue surgery analysis

Plasma samples at baseline and tumor tissue were available for 12 patients. Pathogenic *KRAS* variants were identified in 7 tissue samples; but the same variant in baseline plasma sample was identified in only 2 patients. Regarding *TP53*, pathogenic variants were detected in 3 tissue samples and in 3 baseline plasma samples, although only 1 patient presented the same alteration in tissue and plasma. Besides, 2 patients showed pathogenic alterations in *TP53* only in plasma (Table 2).

**Table 2:** Comparison of genomic alterations identified in plasma basal and tissue samples, only patients with both samples available are included.

	Plasma basal					Tissue				
Patient ID	Altered gene	Variant gene	Variant protein	VAF	Classification	Altered gene	Variant gene	Variant protein	VAF	Classification
P1	None					*CDKN1B* (CNV)	CNV			Pathogenic
P2	***TP53***	**c.537 T > A**	**p.His179Gln**	**0.07**	**Pathogenic**	***TP53***	**c.537 T > A**	**p.His179Gln**	**8.89**	**Pathogenic**
P9	***KRAS***	**c.35G > T**	**p.Gly12Val**	**0.07**	**Pathogenic**	***KRAS***	**c.35G > T**	**p.Gly12Val**	**4.37**	**Pathogenic**
P10	*TP53*	c.992_993insT	p.Gln331HisfsTer6	0.19	Likely pathogenic	None				
P11	*TP53*	c.701A > G	p.Tyr234Cys	0.18	Pathogenic					
						*KRAS*	c.35G > T	p.Gly12Val	17.74	Pathogenic
						*RNF43*	c.1179_1180insT	p.Ala394CysfsTer49	5.27	Likely pathogenic
						*NOTCH2*	c.2755C > T	p.Pro919Ser	49.82	VUS
						*KRAS*	c.35G > A	p.Gly12Asp	11.13	Pathogenic
						*ARID1A*	c.3980_3981insCGCA	p.Gln1327HisfsTer12	7.52	Likely pathogenic
						*POLE*	c.1453A > G	p.Ile485Val	49.61	VUS
P12	None					None				
P14	None					*TP53*	c.842A > G	p.Asp281Gly	20.21	Pathogenic
P13	None					KRAS	c.35G > T	p.Gly12Val	17.42	Pathogenic
P22	*TP53*	c.659A > G	p.Tyr220Cys	0.15	Pathogenic					
	*APC*	c.3920delT	p.Ile1307LysfsTer14	1.1	Likely pathogenic					
						*KRAS*	c.35G > T	p.Gly12Val	19.49	Pathogenic
						*TP53*	c.700 T > A	p.Tyr234Asn	29.93	Likely pathogenic
P23	*TP53*	c.662_663delAG	p.Glu221AlafsTer3	0.08	Likely pathogenic	*TP53*	c.662_663delAG	p.Glu221AlafsTer3	5.97	Likely pathogenic
						*KRAS*	c.35G > A	p.Gly12Asp	4.02	Pathogenic
						*RB1*	c.1850delG	p.Gly617ValfsTer6	6.65	Likely pathogenic
P24	*TP53*	c.992_993insT	p.Gln331HisfsTer6	0.17	Likely pathogenic					
						*TP53*	c.768delA	p.Leu257TrpfsTer88	26.18	Likely pathogenic
						*KRAS*	c.35G > T	p.Gly12Val	14.86	Pathogenic
						*MSH6*	c.2562G > C	p.Lys854Asn	33.89	VUS
						*CCNE1* (CNV)	CNV			Pathogenic
P25	***KRAS***	**c.35G > T**	**p.Gly12Val**	**0.08**	**Pathogenic**	***KRAS***	**c.35G > T**	**p.Gly12Val**	**26.53**	**Pathogenic**
						*TP53*	c.524G > A	p.Arg175His	22.42	Pathogenic

Genomic alterations found in both, plasma and tissue samples are highlighted in bold.

*APC* adenomatous polyposis coli, *ARID1A* AT-rich interaction domain 1A, *CCNE1* cyclin E1, *CDKN1B* cyclin dependent kinase inhibitor 1B, *KRAS* KRAS proto-oncogene, GTPase, *MSH6* mutS homolog6, *NOTCH2* notch receptor 2, *POLE* DNA polymerase epsilon, catalytic subunit, *RB1* RB transcriptional corepresor 1, *RNF43* ring finger protein 43, *TP53* tumor protein p53, VAF variant allele frequency, VUS variant of unknown significance.

### Baseline plasma ctDNA analysis and clinical outcomes

**Figure 3:**
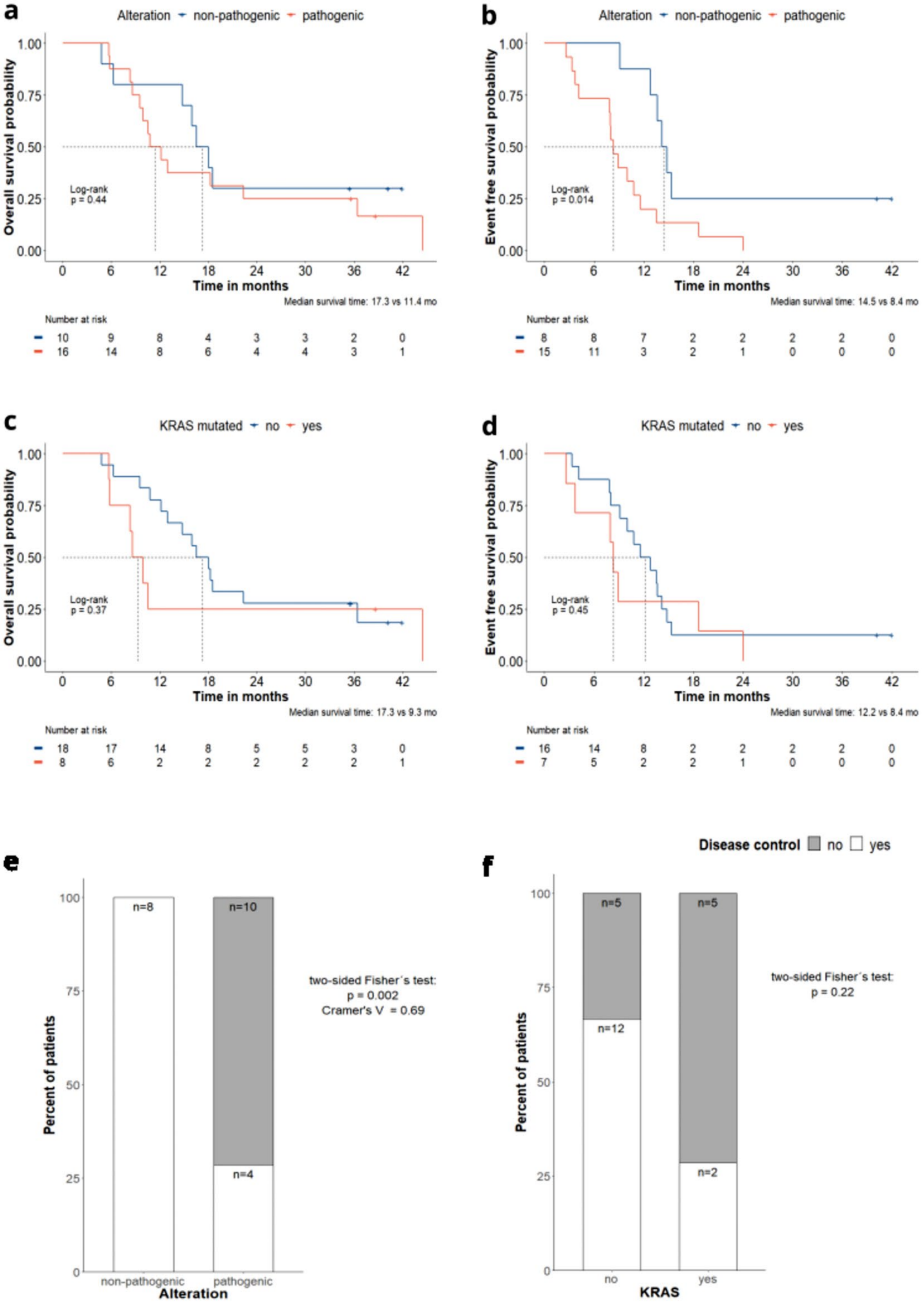
Analyses based on the alterations identified in the baseline plasma sample. Kaplan-Meier analysis comparing patients showing pathogenic alterations (red) *vs* non-pathogenic alterations (i.e., no genetic alteration or alterations identify as non-pathogenic) (blue), (**a**) overall survival (**b**) event free survival. Kaplan-Meier analysis comparing patients showing *KRAS* mutations (red) vs no *KRAS* mutations (blue), (**c**) overall survival (**d**) event free survival. ^**+**^Represents the censored patients. (**e**) Comparison of the proportion of patients with disease control (white) vs no control (grey) in those with pathogenic alterations vs with non-pathogenic alterations (i.e., no genetic alteration or alterations identified as non-pathogenic). (**f**) Comparison of the proportion of patients with disease control (white) vs no control (grey) in those with *KRAS* mutation vs no *KRAS* mutation.

Baseline plasma ctDNA analysis was available for 26 patients, 4 of them with resectable PDAC and 22 with borderline PDAC treated with NAT. Although no significant differences in OS were observed (Fig. 3a), patients with pathogenic alterations in the baseline plasma showed a statistically significant worse EFS than patients with no pathogenic alterations, with a median EFS of 8.4 vs 14 mo, respectively (HR of 3.20; 95% CI: 12.2, 8.5; p = 0.014) (Fig. 3b). When comparing the clinical and pathological parameters of these patients, CA 19-9 levels were higher (p < 0.01) and T4 stage was slightly more represented (not significant) in patients with pathogenic alterations (Table S5). Despite the differences observed in baseline CA 19-9 levels between patients showing pathogenic variants *vs* those not showing pathogenic alterations, CA 19-9 had no effect on EFS analysis when included as an adjusted variable. When assessing the prognostic value of pathogenic *KRAS* variants in these patients, no differences were found neither in OS, nor EFS nor other clinical and pathological parameters between patients harboring pathogenic variants in KRAS variants vs those without pathogenic variants in KRAS in the baseline plasma (Fig. 3c,d, Table S6).

Focusing the 22 borderline PDAC patients treated with NAT, all patients with no pathogenic alterations (n = 8) reached disease control (100%), while only 4 of the 14 patients with pathogenic alterations did reach disease control (28.6%) (p = 0.002) (Fig. 3e). When assessing the association of *KRAS* variants in baseline plasma with disease control, no significant differences in the rate of disease control between patients with and without *KRAS* variants were observed (Fig. 3f).

### First tumor assessment plasma ctDNA analysis and clinical outcomes

Plasma samples at the time of the first tumor assessment post-NAT were available for 23 patients. Pathogenic variants were identified in 9 of these patients (39.1%). No differences were detected in either OS or in EFS, depending on the presence or not of a pathogenic variant in these patients (Fig. S2). *KRAS* criteria was not included in this analysis as the number of patients showing pathogenic *KRAS* variants was too low for comparisons (n = 3).

When considering the response to NAT, the majority of the patients showing no pathogenic variants reached disease control (10 out of 14; 71.4%), while only 4 of the 9 patients showing pathogenic variants reached disease control (44.5%) although this difference was not statiscally significant(Fig. S3).

### Dynamics in variant allele frequency (VAF)

**Figure 4:**
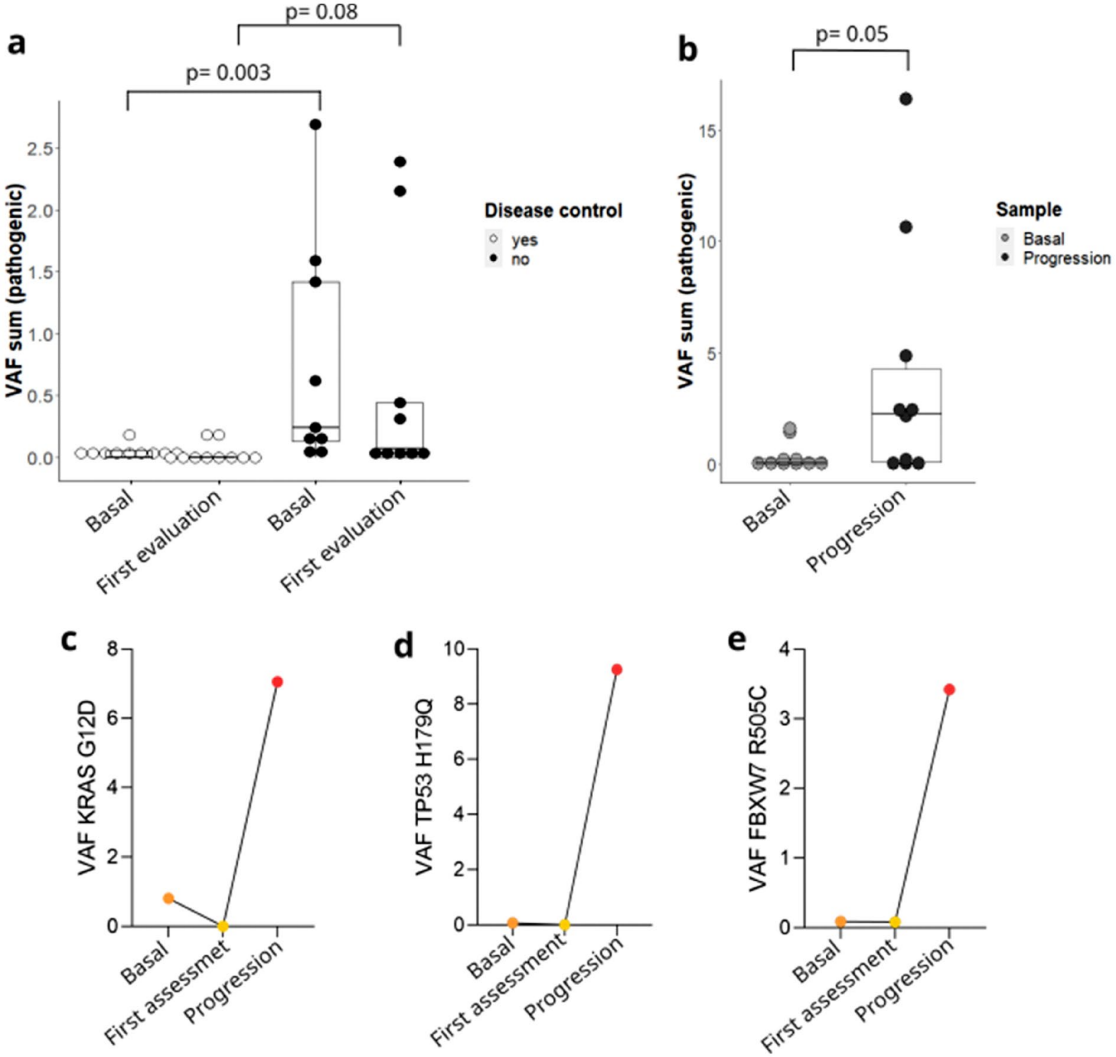
Variant allele frequency changes. Data are represented as with median and interquartile range. (**a**) Comparison in the VAF sum of pathogenic alterations between those with disease control at first tumor assessment (white) and those without (black) in both baseline plasma sample and first tumor assessment plasma sample. (**b**) Comparison in the VAF sum of pathogenic alterations between baseline plasma sample and progression plasma sample. (**c-e**) Dynamics of the VAF in some subjects in which the same alteration was observed in baseline, first tumor assessment and progression plasma samples. All comparisons were performed by Mann-Whitney U test.

Changes related to the frequency of the identified genetic variants, including SNVs, MNVs and short indels were analyzed (i.e., variant allele frequency, VAF). First, the sum of the VAF of the pathogenic variants of both baseline plasma and first tumor assessment plasma were compared in patients reaching disease control or not (Fig. 4a). The VAF sum in baseline plasma was significantly higher in patients that did not reach disease control compared to those that reached disease control (p = 0.003), a similar trend, although not statistically significant, was observed in the plasma taken at first tumor assessment. When analyzing dynamics from baseline plasma to progression, overall VAF was higher at progression than in the baseline sample (p = 0.05) (Fig. 4b). In some patients having baseline, first tumor assessment and progression plasma samples available, VAF dynamics of selected variants were followed. In this regard, one patient showed two variants that could be followed in genes *KRAS* and *FBXW7* and in another patient, a variant in *TP53* could be followed as well. The variants in *KRAS* and *TP53* were identified in the baseline plasma, disappeared after neoadjuvant treatment and were detected again at a higher VAF at progression (Fig. 4c,d). The variant in *FBX7* showed the same tendency, albeit it was still detectable in first tumor assessment plasma at a lower VAF than in the baseline plasma (Fig. 4e).

## Discussion

PDAC ranks the 14th cause of cancer but represents the 7th leading cause of cancer-related death^3^, thus reflecting its inherent aggressiveness. On top of this, PDAC incidence has been increasing in the last decades and it is projected to represent one of the main cancers in the upcoming years^4^. Patients with PDAC have an impaired outcome, and limited therapeutic options are currently available^27^. In this scenario, personalized medicine appears as a desirable option to enhance prognosis for PDAC patients, and the incorporation of ctDNA analyses in the routine therapeutic armamentarium arising as a potential complementary tool.

The utility of ctDNA analysis is sustained on the tumor dynamics^13,24^. ctDNA is released in peripheral blood from tumor cells undergoing apoptosis, necrosis or even due to metabolic secretion. Advances in genome sequencing including targeted methods such as NGS have made analysis of ctDNA feasible for clinical use. Accumulating evidence supports this approach at different time points along disease progression, including early diagnosis, detection of minimal residual disease after surgery, molecular characterization for selection of targeted therapy, and monitoring treatment response. Although advances have achieveddone in lung, breast and colon cancer, evidence is less advanced in PDAC and needs further translation to the clinics^13,14,24^.

More than 90% of PDAC present *KRAS* variants, the most frequently mutated oncogene in cancer^28^. Although not targetable for many years, novel approaches have been developed for mutant *KRAS* tumors in the last decade, thus making some mutated variants of this gene druggable^28,29^. On the other hand, non-mutant *KRAS* PDAC shows a greater number of molecular alterations with potential value for precision medicine. For example, pathogenic germline variants in different genes, such as *BRCA1, BRCA2* or *PALB2,* or somatic-level fusions in *NRG1*, rearranged *RET* or *NTRK*^30–33^.

Consistent with the literature, we found *KRAS* and *TP53* as the most frequently mutated genes. While the genetic landscape described in tumor tissue was consistent to what had been previously reported (87.5% of patients presented pathogenic alterations, mostly variants in *KRAS* (81.3%), plasma ctDNA results only partially covered this scenario. Percentages of patients showing pathogenic alterations varied according to different time-points: 61.5% in baseline plasma samples, 39.1% in first tumor assessment plasma and 80% in progression plasma, thus reflecting the dynamics of tumor burden. Also, proportions of each mutation changed in time. These differences probably reflect tumor heterogeneity and clonal evolution. When comparing tissue and baseline plasma analyses, plasma analyses could not detect pathogenic alterations in a percentage of the patients, which did indeed present alterations in their tissue. Previous studies assessing concordance levels between tissue and ctDNA NGS in gastrointestinal cancers have reported varying results depending on the experimental settings^19,21,34,35^. This phenomenon could be explained due to the small amounts of ctDNA present in blood at certain settings, including early stage and non-shedding tumors^12,36^. Additionally, these results can also be expected given the differences between the employed sequencing panels; i.e. the plasma panel (Oncomine™ Pan-cancer Cell-Free Assay) does not cover some of the genetic regions sequenced by the tissue panel (Oncomine™ Comprehensive v3 Assay). In summary, our results suggest that in the non-metastasic setting of PDAC, tissue-biopsy should remain as the gold standard to characterize tumor genetics, while ctDNA analysis are still investigational. Possible methods to overcome its sensibility and sensitivity may consider the collection of larger amounts of plasma. Nevertheless, we could show how plasma ctDNA analysis plays a complementary role to tissue-biopsy, by improving molecular characterization. Moreover, baseline plasma analyses revealed pathogenic variants in cancer-related genes beyond *KRAS* and *TP53* in patients with no tissue samples available. Indeed, ctDNA was suggested to be more informative in the metastatic setting, being able to recover the genetic heterogeneity derived from different lesions, some of them potentially druggable, therefore identifying candidates for treatment with targeted therapies^35^.

Besides its value for genetic testing, baseline ctDNA analysis in the non-metastatic PDAC holds promise as a prognostic tool to identify patients with poor clinical outcomes as suggested by recent studies in the field^16,22,37–39^. In line with this, we were able to demonstrate a poorer outcome in patients with pathogenic alterations in baseline plasma, compared to those with non-pathogenic alterations (i.e., patients presenting no genetic alterations or only VUS or likely pathogenic variants). When focusing on *KRAS* variants, patients did not differ in prognosis, although this was probably related to the low number of *KRAS* mutated patients.

Resectability of non-metastatic PDAC patients mainly relies on the tumor vascular invasion. In patients presenting borderline PDAC^7,8^, induction chemotherapy treatment is recommended, with the aim of eradicating micrometastatic disease and down staging the tumor, and with the intention of attempting secondary curative surgery^1,27^. In this scenario, ctDNA analysis in baseline and after NAT blood samples could serve a complementary role to current strategies of response assessment^40,41^.Our results build on this direction, as we could observe how all the patients with borderline PDAC with non-pathogenic alterations in baseline plasma reached disease control after NAT, thus being suitable for surgery. By contrast, only the 28.6% of the patients with pathogenic alterations reached disease control after NAT (p = 0.002). When considering the results on the ctDNA analyses performed after the neoadjuvant treatment and prior to surgery (at the first tumor assessment), patients with non-pathogenic alterations tend to present higher rates of disease control than patients with pathogenic alterations, although this difference was not statically significant.

Type of neoadjuvant chemotherapy is still a matter of debate^9,10^. Considering the purpose of this approach, identifying genetic alterations to guide treatment decisions would be crucial. Preference of platinum combinations in patients with mutations in the homologous recombination genes^11^ or targeted therapies in patients with druggable mutations would enhance treatment results. We could observe some pathogenic variants in different cancer-associated genes including *BRAF*, *FGFR2* or *ERBB3* that, although not currently available in the clinical practice, may be considered for treatment in future clinical trials.

Finally, higher VAF of pathogenic genetic variants in circulating tumor DNA is an indicator of poor outcome^42^. We could observe how patients with baseline plasma samples showing higher VAF of pathogenic variants presented a worse prognosis than patients with lower VAFs. A tendency was shown when considering samples taken at the end of the neoadjuvant treatment and prior to surgery. Similarly, the VAF sum was greater at the progression plasma samples, reflecting the progression of the disease, in agreement with previous studies, suggesting that ctDNA detection associates with tumor mutational load^17,36,43^. Specifically, we were able to demonstrate the utility of VAF dynamics in two patients, in which *KRAS* and *TP53* variants from plasma samples were longitudinally followed. They were detected in baseline plasma, disappeared after neoadjuvant treatment prior to surgery, and were again detected at a higher VAF in the sample taken at progression. Pathogenic alterations reflect the tumor mutational load and can be used to monitor treatment response and to identify resistance mutations at progression^42,44,45^.

Our study has some limitations, including the small number of enrolled patients. We used two different sequencing panels: the Oncomine™ Pan-cancer Cell-Free Assay for plasma samples analysis and Oncomine™ Comprehensive v3 Assay for tumor tissue. The Oncomine™ Comprehensive covers 161 cancer-related genes, while Oncomine™ Pan-cancer is designed to detect alterations in 52 genes, meaning that pathogenic genetic alterations in other genes may not have been detected. Additionally, post-surgical samples were missed, thus impeding the evaluation of minimal residual disease in this setting.

## Conclusions

In conclusion, this study represents the first approach to ctDNA analysis in PDAC in our hospital. This study is part of a granted research program for the development of precision medicine in Navarra, Spain. We have been able to build on the potential value of ctDNA analysis for molecular profile characterization, monitoring therapy response, detection of tumor relapse and prognosis in PDAC. All these findings are in line with the current literature and build on the clinical applicability of ctDNA analysis in non-metastatic PDAC. However, prospective studies are needed to define the appropriate way to use ctDNA as a tool in the clinic.

### Supplementary Information

Supplementary Information.

## Data Availability

The data underlying this article cannot be shared publicly due to ethical restrictions. The data will be shared on reasonable request to the corresponding author.
