# Supplementary material for: Building on the clinical applicability of ctDNA analysis in non-metastatic pancreatic ductal adenocarcinoma
Source: Sci Rep. 2024 Jul 13;14:16203. doi: 10.1038/s41598-024-67235-y (PMC11246447; doi:10.1038/s41598-024-67235-y)
Supplement: Supplementary file 1 — Supplementary Information. [file 41598_2024_67235_MOESM1_ESM.docx]

# **Supplementary information**

**Tittle:** **Building on the clinical applicability of ctDNA analysis in non-metastatic pancreatic ductal adenocarcinoma**

Ibone Labiano^1^, Ana E Huerta^1^, Maria Alsina*^1,2^, Hugo Arasanz^1,2^, Natalia Castro^1,^, Saioa Mendaza^3^, Arturo Lecumberri^1,2^, Iranzu Gonzalez-Borja^1^, David Guerrero^5^, Ana Patiño-Garcia^6^, Gorka Alkorta^7^, Irene Hernández-Garcia^1,2^, Virginia Arrazubi^1,2^, Elena Mata^1,2^, David Gomez^1,2^, Antonio Viudez^1,8^, Ruth Vera^1,2^.

*Corresponding author. Email: [maria.alsina.maqueda@navarra.es](mailto:maria.alsina.maqueda@navarra.es). ORCID: 0000-0003-4835-7159

**Elements included in the document:**

**Fig. S1.** Frequency of patients presenting pathogenic genomic alterations by gene

**Fig. S2.** Survival analyses based on the alterations identified in the first tumor assessment plasma sample

**Fig. S3.** First tumor assessment plasma alteration analysis according with disease control

**Table S1**: Genomic alterations identified for each patients in basal plasma samples

**Table S2**: Genomic alterations identified for each patients in basal first evaluation plasma samples.

**Table S3**: Genomic alterations identified for each patients in basal progression plasma samples

**Table S4**: Genomic alterations identified for each patients in tissue samples

**Table S5**: Comparison of the clinico-pathological parameters of the patients according to the presence of pathogenic variants on their basal plasma samples

**Table S6**: Comparison of the clinico-pathological parameters of the patients according to the presence of KRAS mutations on their basal plasma samples available


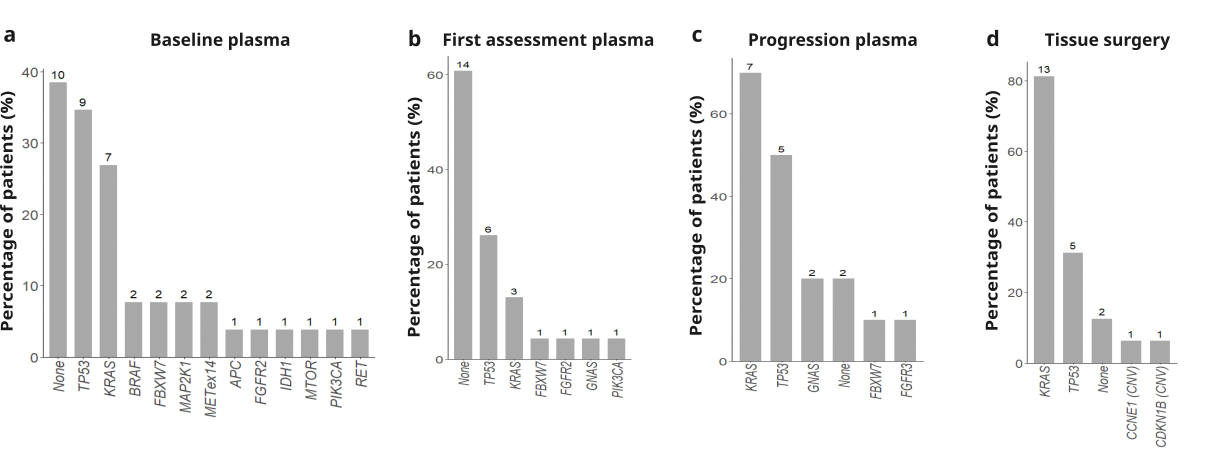


**Fig. S1. Frequency of patients presenting pathogenic genomic alterations by gene. a)** Percentage and number of patients showing pathogenic genomic alterations for each gene in baseline plasma samples. **b)** Percentage and number of patients showing pathogenic genomic alterations for each gene in first tumor assessment plasma samples. **c)** Percentage and number of patients showing pathogenic genomic alterations for each gene in progression plasma samples. **d)** Percentage and number of patients showing pathogenic genomic alterations for each gene in progression plasma samples.


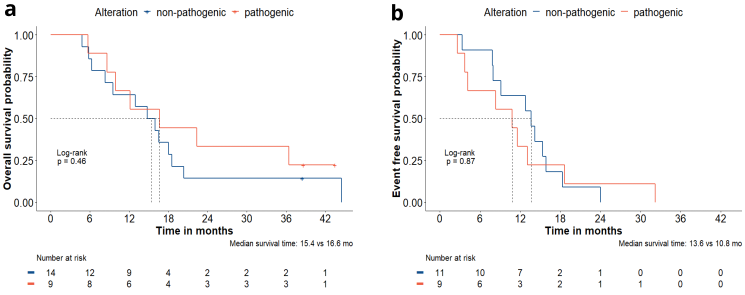


**Fig. S2. Survival analyses based on the alterations identified in the first tumor assessment plasma sample.** Kaplan-Meier analysis comparing patients showing pathogenic alterations (red) *vs* non-pathogenic alterations (i.e., no genetic alteration or alterations identified as non-pathogenic) (blue) **a)** overall survival, **b)** event free survival.

+Represents the censored patients.


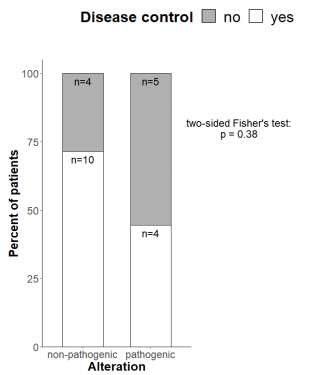


**Fig. S3. First tumor assessment plasma alteration analysis according with disease control.** Comparison of the proportion of patients with disease control (white) vs no control (grey) in those patients with pathogenic alterations vs non-pathogenic alterations (i.e., no genetic alteration or alterations identified as non-pathogenic).

| Table S1. Genomic alterations identified for each patients in basal plasma samples | | | | | |  | |  | |
| --- | --- | --- | --- | --- | --- | --- | --- | --- | --- |
| PATIENT  ID | **Altered**  **gene** | **Variant**  **gene** | **Variant**  **protein** | **VAF** | **Classification** | **COSMIC (number of patients with pancreatic cancer harboring each alteration, and other malignancies harboring the same alteration)** | | **CHIP (alteration identified as CHIP)** | |
| P1 | None |  |  |  |  |  | |  | |
| P2 | *TP53* | c.537T>A | p.His179Gln | 0.07 | Pathogenic | Pancreas 0 (breast, large intestine, lung,hematopoietic and lymphoid) | | No | |
| P3 | *TP53* | c.776A>T | p.Asp259Val | 0.17 | Pathogenic | Pancreas 3 | | No | |
| P4 | *TP53* | c.470T>C | p.Val157Ala | 0.11 | Pathogenic | Pancreas 0 (esophagous, Large intenstine,biliary tract, liver, hematopoietic and lymphoid) | | No | |
|  | *TP53* | c.437G>A | p.Trp146* | 0.16 | Pathogenic | Pancreas 6 | | No | |
|  | *TP53* | c.443A>G | p.Asp148Gly | 0.16 | Uncertain | Pancreas 0 (lung) | | No | |
|  | *TP53* | c.1101-1G>A | p.? | 0.13 | Pathogenic | Pancreas 0 (lung, hematopoietic and lymphoid) | | No | |
|  | *KRAS* | c.35G>A | p.Gly12Asp | 0.8 | Pathogenic | Pancreas 3544 | | No | |
|  | *APC* | c.4393_4394delAG | p.Ser1465Trpfs | 0.13 | Pathogenic | No | | No data | |
|  | *FBXW7* | c.1513C>T | p.Arg505Cys | 0.09 | Pathogenic | Pancreas 0 (large intestine, endometrium, upper aerodigestive tract, skin, hematopoietic and lymphoid) | | No | |
|  | *MET*ex14 | RNA exon variant |  |  | Pathogenic | - | | No data | |
| P5 | *MET*ex14 | RNA exon variant |  |  | Pathogenic | - | | No data | |
|  | *TP53* | c.943delT | p.Ser315LeufsTer30 | 0.15 | Likely pathogenic | No data | | No | |
| P6 | *TP53* | c.992_993insT | p.Gln331HisfsTer6 | 0.23 | Likely pathogenic | No data | | No | |
| P7 | *TP53* | c.272G>A | p.Trp91* | 0.96 | Pathogenic | Pancreas 4 | | No | |
|  | *KRAS* | c.34G>C | p.Gly12Arg | 0.63 | Pathogenic | Pancreas 958 | | No | |
| P8 | *KRAS* | c.34G>C | p.Gly12Arg | 0.24 | Pathogenic | Pancreas 958 | | No | |
| P9 | *KRAS* | c.35G>T | p.Gly12Val | 0.07 | Pathogenic | Pancreas 2404 | | M-CHIP | |
| P10 | *TP53* | c.992_993insT | p.Gln331HisfsTer6 | 0.19 | Likely pathogenic | No data | | No | |
| P11 | *TP53* | c.701A>G | p.Tyr234Cys | 0.18 | Pathogenic | Pancreas 0 (lung, breast, large intestine, central nervous system, hematopoietic and lymphoid) | | No | |
| P12 | None |  |  |  |  |  | |  | |
| P13 | None |  |  |  |  |  | |  | |
| P14 | None |  |  |  |  |  | |  | |
| P15 | None |  |  |  |  |  | |  | |
| P16 | *KRAS* | c.35G>T | p.Gly12Val | 0.05 | Pathogenic | Pancreas 2404 | | M-CHIP | |
|  | *IDH1* | c.395G>A | p.Arg132His | 0.57 | Pathogenic | Pancreas 0 (central nervous system, bone, biliary tract, prostate) | | No | |
| P17 | *BRAF* | c.1781A>G | p.Asp594Gly | 0.09 | Pathogenic | Pancreas 0 (large intestine, lung, skin,endometrium, hematopoietic and lymphoid) | | No | |
| P18 | *FGFR2* | c.1975A>G | p.Lys659Glu | 0.13 | Pathogenic | No data | | No data | |
| P19 | *TP53* | c.586C>T | p.Arg196* | 0.74 | Pathogenic | Pancreas 0 (large intestine, breast, esophagous, skin, stomach) | | No | |
|  | *KRAS* | c.35G>A | p.Gly12Asp | 1.95 | Pathogenic | Pancreas 3544 | | No | |
| P20 | None |  |  |  |  |  | |  | |
| P21 | *TP53* | c.517G>A | p.Val173Met | 0.07 | Pathogenic | Pancreas 0 (large intestine, breast, esophagous, upper aerodigestive tract, hematopoietic and lymphoid) | | No | |
| P22 | *TP53* | c.659A>G | p.Tyr220Cys | 0.15 | Pathogenic | Pancreas 0 (breast, ovary, lung, esophagus, hematopoietic and lymphoid) | | No | |
|  | *APC* | c.3920delT | p.Ile1307LysfsTer14 | 1.1 | Likely pathogenic | No data | | No data | |
| P23 | *TP53* | c.662_663delAG | p.Glu221fs | 0.08 | Likely pathogenic | No data | | No | |
| P24 | *TP53* | c.992_993insT | p.Gln331HisfsTer6 | 0.17 | Likely pathogenic | No data | | No | |
| P25 | *KRAS* | c.35G>T | p.Gly12Val | 0.08 | Pathogenic | Pancreas 2404 | | M-CHIP | |
| P30 | *TP53* | c.455_456insC | p.Pro153AlafsTer28 | 0.48 | Pathogenic | No data | | No | |
|  | *TP53* | c.829T>C | p.Cys277Arg | 0.15 | Pathogenic | Pancreas 0 (large intestine, central nervous system) | | No | |
|  | *TP53* | c.755T>C | p.Leu252Pro | 0.1 | Pathogenic | Pancreas 0 (lung, skin, large intestine, ovary, breast) | | No | |
|  | *TP53* | c.721T>C | p.Ser241Pro | 0.1 | Pathogenic | Pancreas 1 (large intestine, skin, prostate, hematopoietic and lymphoid) | | No | |
|  | *TP53* | c.586C>T | p.Arg196* | 0.09 | Pathogenic | Pancreas 0 (large intestine, breast, esophagus, skin, stomach) | | No | |
|  | *TP53* | c.470T>C | p.Val157Ala | 0.13 | Pathogenic | Pancreas 0 (esophagous, large intestine, biliary tract, liver, hematopoietic and lymphoid) | | No | |
|  | *TP53* | c.438G>A | p.Trp146* | 0.09 | Pathogenic | Pancreas 0 (large intestine, breast, esophagous, lung, upper aerodigestive tract) | | No | |
|  | *TP53* | c.97-2A>G | p.Cys141Arg | 0.39 | Pathogenic | Pancreas 0 (central nervous system, prostate, lung, upper aerodigestive tract, hematopoietic and lymphoid) | | No | |
|  | *FBXW7* | c.1513C>T | p.Arg505Cys | 0.28 | Pathogenic | Pancreas 0 (large intestine, endometrium, upper aerodigestive tract, skin, hematopoietic and lymphoid) | | No | |
|  | *PIK3CA* | c.3073A>G | p Thr1025Ala | 0.26 | Pathogenic | Pancreas 0 (endometrium, large intestine, breat, stomach,thyroid) | | No data | |
|  | *MAP2K1* | c.157T>C | p.Phe53Leu | 0.2 | Pathogenic | Pancreas 0 (urinary tract, large intestine, lung, stomach, hematopoietic and lymphoid) | | No | |
|  | *MAP2K1* | c.169A>G | p.Lys57Glu | 0.13 | Pathogenic | Pancreas 0 (large intestiene, breast, biliary tract, small intestine, hematopoietic and lymphoid) | | No | |
|  | *BRAF* | c.1799 T>C | p.Val600Ala | 0.13 | Pathogenic | Pancreas 0 (prostate, large intestine, skin) | | No | |
|  | *FGFR2* | c.1144T>C | p.Cys382Arg | 0.2 | Likely pathogenic | No data | | No data | |
|  | *MTOR* | c.6040G>A | p.Glu2014Lys | 0.15 | VUS | Pancreas 0 (urinary tract, large intestine) | | No data | |
|  | *MTOR* | c.6643T>C | p.Ser2215Pro | 0.15 | Pathogenic | Pancreas 0 (genital tract) | | No | |
|  | *ESR1* | c.1387T>C | p.Ser463Pro | 0.12 | VUS | Pancreas 0 (breast, endometrium) | | No data | |
|  | *RET* | c.1900T>C | p.Cys634Arg | 0.07 | Pathogenic | Pancreas 0 (thyroid, adrenal gland) | | No | |
|  | *AR* | c.2632A>G | p.Thr878Ala | 0.27 | Likely pathogenic | Pancreas 0 (prostate, liver) | | No data | |
| Abbreviations: *APC*: adenomatous polyposis coli; *AR*: androgen receptor; CHIP: clonal hematopoiesis of indeterminate potential; COSMIC: catalogue of somatic mutations in cancer; *BRAF*: B-Raf proto-oncogene. serine/threonine kinase; *ESR1*: estrogen receptor 1; *FBXW7*: F-Box And WD Repeat Domain Containing 7; *FGFR2*: fibroblast growth factor receptor 2; *KRAS*: KRAS proto-oncogene. GTPase; *IDH1*: isocitrate dehydrogenase (NADP(+)) 1; *MAP2K1*: mitogen-activated protein kinase kinase 1; METex14: MET proto-oncogene. receptor tyrosine kinase skipping of exon 14; *MTOR*: mechanistic target of rapamycin kinas; *PIK3CA*: phosphatidylinositol-4.5-bisphosphate 3-kinase catalytic subunit alpha; *TP53*: tumor protein p53; *RET*: ret proto-oncogene; VAF: variant allele frequency; VUS: variant of unknown significance. | | | | | | |  | |  |

**Table S2**. Genomic alterations identified for each patients in first evaluation plasma samples.

| **Patient**  **ID** | **Altered**  **gene** | **Variant**  **gene** | **Variant**  **protein** | **VAF** | **Classification** | **COSMIC (number of patients with pancreatic cancer harboring each alteration, and other malignancies harboring the same alteration)** | **CHIP (alteration identified as CHIP)** |
| --- | --- | --- | --- | --- | --- | --- | --- |
| **P1** | None |  |  |  |  |  |  |
| **P2** | None |  |  |  |  |  |  |
| **P3** | *GNAS* | c.602G>A | p.Arg201His | 0.07 | Pathogenic | No data | No |
| **P4** | *TP53* | c.709A>G | p.Met237Val | 0.13 | Pathogenic | Pancreas 0 (large intestine, breast,upper aerodigestive tract, lung, hematopoietic and lymphoid) | No |
|  | *TP53* | c.718A>G | p.Ser240Gly | 0.1 | Pathogenic | Pancreas 0 (large intestine, soft tissue, lung, central nervous system, hematopoietic and lymphoid) | No |
|  | *TP53* | c.691A>G | p.Thr231Ala | 0.08 | VUS | Pancreas 0 (prostate, thyroid) | No |
|  | *TP53* | c.662A>G | p.Glu221Gly | 0.09 | VUS | Pancreas 0 (large intestine, lung, skin) | No |
|  | *FBXW7* | c.1513C>T | p.Arg505Cys | 0.08 | Pathogenic | Pancreas 0 (large intestine, endometrium, upper aerodigestive tract, skin, hematopoietic and lymphoid) | No |
|  | *PIK3CA* | c.1633G>A | p.Glu545Lys | 0.13 | Pathogenic | Pancreas 0 (breast, large intestinte, urinary tract, upper aerodigestive tract, cervix) | No data |
|  | *PTEN* | c.1028T>C | p.Val343Ala | 0.08 | VUS | Pancreas 0 (skin) | No data |
|  | *PDGFRA* | c.1736A>G | p.Gln579Arg | 0.16 | VUS | No data | No data |
| **P5** | None |  |  |  |  |  |  |
| **P6** | None |  |  |  |  |  |  |
| **P7** | *TP53* | c.272G>A | p.Trp91* | 1.32 | Pathogenic | Pancreas 4 | No |
|  | *KRAS* | c.34G>C | p.Gly12Arg | 1.07 | Pathogenic | Pancreas 958 | No |
| **P8** | *TP53* | c.916C>T | p.Arg306* | 0.96 | Pathogenic | Pancreas 0 (large intestine, breast, esophagous, upper aerodigestive tract, stomach) | No |
|  | *KRAS* | c.34G>C | p.Gly12Arg | 1.19 | Pathogenic | Pancreas 958 | No |
| **P9** | *KRAS* | c.35G>T | p.Gly12Val | 0.18 | Pathogenic | Pancreas 2404 | M-CHIP |
| **P10** | None |  |  |  |  |  |  |
| **P11** | *TP53* | c.701A>G | p.Tyr234Cys | 0.18 | Pathogenic | Pancreas 0 (lung, breast, large intestine, central nervous system, hematopoietic and lymphoid) | No |
| **P12** | None |  |  |  |  |  |  |
| **P13** | *TP53* | c.992_993insT | p.Gln331HisfsTer6 | 1.75 | Likely pathogenic | No data | No |
| **P15** | None |  |  |  |  |  |  |
| **P16** | None |  |  |  |  |  |  |
| **P17** | None |  |  |  |  |  |  |
| **P18** | *FGFR2* | c.1975A>G | p.Lys659Glu | 0.31 | Pathogenic | No data | No data |
| **P19** | None |  |  |  |  |  |  |
| **P20** | None |  |  |  |  |  |  |
| **P26** | *TP53* | c.747G>C | p.Arg249Ser | 0.08 | Pathogenic | Pancreas 0 (lung, liver,breast, large intestine, urinary tract) | No |
| **P27** | *TP53* | c.818G>A | p.Arg273His | 0.07 | Pathogenic | Pancreas 0 (large intestine, breast, lung, central nervous system, ovary) | No |
| **P28** | None |  |  |  |  |  |  |
| **P29** | *TP53* | c.742C>G | p.Arg248Gly | 0.11 | Likely pathogenic | Pancreas 0 (lung, breast, ovary, large intestinte, hematopoietic and lymphoid) | No |

Abbreviations: CHIP: clonal hematopoiesis of indeterminate potential; COSMIC: catalogue of somatic mutations in cancer; *FBXW7*: F-Box And WD Repeat Domain Containing 7; *FGFR2*: fibroblast growth factor receptor 2; GNAS: *GNAS* complex locus; *KRAS*: KRAS proto-oncogene. GTPase; *PIK3CA*: phosphatidylinositol-4.5-bisphosphate 3-kinase catalytic subunit alpha; *PDGFRA*: Platlet-derived growth factor receptor alpha; *PTEN*: phosphatase and tensin homolog; *TP53*: tumor protein p53; *RET*: ret proto-oncogene; VAF: variant allele frequency; VUS: variant of unknown significance.

| **Table S3**: Genomic alterations identified for each patient in the progression plasma sample | | | | | |  |  |
| --- | --- | --- | --- | --- | --- | --- | --- |
| **Patient**  **ID** | **Altered**  **gene** | **Variant**  **gene** | **Variant**  **protein** | **VAF** | **Classification** | **COSMIC (number of patients with pancreatic cancer harboring each alteration, and other malignancies harboring the same alteration)** | **CHIP (alteration identified as CHIP)** |
| **P1** | *KRAS* | c.35G>A | p.Gly12Val | 0.2 | Pathogenic | Pancreas 3544 | No |
| **P2** | *TP53* | c.537T>A | p.His179Gln | 9.25 | Pathogenic | Pancreas 0 (breast, large intestine, lung, stomach, hematopoietic and lymphoid) |  |
|  | *KRAS* | c.35G>A | p.Gly12Val | 7.16 | Pathogenic | Pancreas 3544 | No |
| **P3** | *GNAS* | c.602G>A | p.Arg201His | 0.07 | Pathogenic | No data | No |
| **P4** | *TP53* | c.821T>C | p.Val274Ala | 0.08 | Likely pathogenic | Pancreas 0 (large intestine, breast, prostate,upper aerodigestive tract, liver) | No |
|  | *TP53* | c.274C>G | p.Pro92Ala | 0.28 | VUS | Pancreas 0 (hematopoietic and lymphoid) | No |
|  | *KRAS* | c.35G>A | p.Gly12Asp | 7.06 | Pathogenic | Pancreas 3544 | No |
|  | *GNAS* | c.601C>T | p.Arg201Cys | 0.07 | Pathogenic | No data | No |
|  | *FBXW7* | c.1513C>T | p.Arg505Cys | 3.42 | Pathogenic | Pancreas 0 (large intestine, endometrium, upper aerodigestive tract, skin, hematopoietic and lymphoid) | No |
|  | *FGFR3* | c.1948A>G | p.Arg650Glu | 0.1 | Pathogenic | No data | No data |
|  | *MET* | c.3802A>G | p.Met1268Val | 0.1 | Likely pathogenic | No data | No data |
| **P5** | None |  |  |  |  |  |  |
| **P6** | *TP53* | c.455_456insC | p.Pro153AlafsTer28 | 1.3 | Pathogenic | No data | No |
|  | *TP53* | c.752T>C | p.Ile251Thr | 0.09 | Pathogenic | Pancreas 0 (large intestine, stomach, hematopoietic and lymphoid) | No |
|  | *TP53* | c.745A>T | p.Arg249Trp | 0.09 | Likely pathogenic | Pancreas 0 (lung, breast, large intestine, esophagous, urinary tract) | No |
|  | *KRAS* | c.35G>A | p.Gly12Asp | 1.08 | Pathogenic | Pancreas 3544 | No |
| **P7** | *TP53* | c.272G>A | p.Trp91* | 1.32 | Pathogenic | Pancreas 4 | No |
|  | *KRAS* | c.34G>C | p.Gly12Arg | 1.07 | Pathogenic | Pancreas 958 | No |
| **P8** | *TP53* | c.916C>T | p.Arg306* | 0.96 | Pathogenic | Pancreas 0 (large intestine, breast, esophagous,upper aerodigestive tract, stomach) | No |
|  | *KRAS* | c.34G>C | p.Gly12Arg | 1.19 | Pathogenic | Pancreas 958 | No |
| **P14** | None |  |  |  |  |  |  |
| **P21** | *TP53* | c.517G>A | p.Val173Met | 0.07 | Pathogenic | Pancreas 0 (large intestine, breast, esophagous, upper aerodigestive tract, hematopoietic and lymphoid) | No |
|  | *TP53* | c.709A>G | p.Met237Val | 0.79 | Pathogenic | Pancreas 0 (large intestine, breast, upper aerodigestive tract, lung) | No |
|  | *KRAS* | c.183A>C | p.Gln61His | 3.99 | Pathogenic | Pancreas 82 | No |
| Abbreviations: CHIP: clonal hematopoiesis of indeterminate potential; COSMIC: catalogue of somatic mutations in cancer; FBXW7: F-Box and WD Repeat Domain Containing 7; FGFR3: fibroblast growth factor receptor 3; GNAS: GNAS complex locus; MET proto-oncogene; Abbreviations: CHIP: clonal hematopoiesis of indeterminate potential; COSMIC: catalogue of somatic mutations in cancer; FBXW7: F-Box And WD Repeat Domain Containing 7; FGFR3: fibroblast growth factor receptor 3; GNAS: GNAS complex locus; MET proto-oncogene; KRAS: KRAS proto-oncogene. GTPase; TP53: tumor protein p53; VAF: variant allele frequency; VUS: variant of unknown significance. | | | | | |  |  |

| **Table S4**: Genomic alterations identified for each patients in tissue samples | | | | | |
| --- | --- | --- | --- | --- | --- |
| **Patient**  **ID** | **Altered**  **gene** | **Variant**  **gene** | **Variant**  **protein** | **VAF** | **Classification** |
| **P1** | *CDKN1B* (CNV) | CNV |  |  | Pathogenic |
| **P2** | *TP53* | c.537T>A | p.His179Gln | 8.89 | Pathogenic |
|  | *KRAS* | c.35G>A | p.Gly12Asp | 6.37 | Pathogenic |
|  | *ARID1A* | c.2627_2628insCC | p.Pro877HisfsTer15 | 6.96 | Likely pathogenic |
| **P9** | *KRAS* | c.35G>T | p.Gly12Val | 4.37 | Pathogenic |
|  | *RNF43* | c.883delC | p.His295IlefsTer124 | 7.15 | Likely pathogenic |
| **P10** | None |  |  |  |  |
| **P11** | *KRAS* | c.35G>A | p.Gly12Asp | 11.13 | Pathogenic |
|  | *ARID1A* | c.3980_3981insCGCA | p.Gln1327HisfsTer12 | 7.52 | Likely pathogenic |
|  | *POLE* | c.1453A>G | p.Ile485Val | 49.61 | VUS |
| **P12** | None |  |  |  |  |
| **P13** | *KRAS* | c.35G>T | p.Gly12Val | 17.42 | Pathogenic |
| **P14** | *TP53* | c.842A>G | p.Asp281Gly | 20.21 | Pathogenic |
|  | *KRAS* | c.35G>T p:e-161;354 | p.Gly12Val | 17.74 | Pathogenic |
|  | *RNF43* | c.1179_1180insT | p.Ala394CysfsTer49 | 5.27 | Likely pathogenic |
|  | *NOTCH2* | c.2755C>T | p.Pro919Ser | 49.82 | VUS |
| **P22** | *KRAS* | c.35G>T | p.Gly12Val | 19.49 | Pathogenic |
|  | *TP53* | c.700T>A | p.Tyr234Asn | 29.93 | Likely pathogenic |
| **P23** | *TP53* | c.662_663delAG | p.Glu221AlafsTer3 | 5.97 | Likely pathogenic |
|  | *KRAS* | c.35G>A | p.Gly12Asp | 4.02 | Pathogenic |
|  | *RB1* | c.1850delG | p.Gly617ValfsTer6 | 6.65 | Likely pathogenic |
| **P24** | *TP53* | c.768delA | p.Leu257TrpfsTer88 | 26.18 | Likely pathogenic |
|  | *KRAS* | c.35G>T | p.Gly12Val | 14.86 | Pathogenic |
|  | *MSH6* | c.2562G>C | p.Lys854Asn | 33.89 | VUS |
|  | *CCNE1* (CNV) | CNV |  |  | Pathogenic |
| **P25** | *TP53* | c.524G>A | p.Arg175His | 22.42 | Pathogenic |
|  | *KRAS* | c.35G>T | p.Gly12Val | 26.53 | Pathogenic |
| **P26** | *KRAS* | c.35G>T | p.Gly12Val | 10.82 | Pathogenic |
| **P27** | *KRAS* | c.35G>T | p.Gly12Val | 3.81 | Pathogenic |
|  | *ERBB3* | c.850G>A | p.Gly284Arg | 9.5 | VUS |
| **P28** | *KRAS* | c.34G>C | p.Gly12Arg | 10.36 | Pathogenic |
|  | *TP53* | c.817C>T | p.Arg273Cys | 5.55 | Pathogenic |
|  | *ATRX* | c.430C>T | p.Pro144Ser | 5.96 | VUS |
|  | *FANCD2* | c.688G>A | p.Glu230Lys | 5.59 | VUS |
|  | *ERBB4* | c.2398C>T | p.Pro800Ser | 5.07 | VUS |
| **P29** | *KRAS* | c.182A>G | p.Gln61Arg | 7.45 | Pathogenic |
|  | *TP53* | c.736A>C | p.Met246Leu | 12.05 | Pathogenic |
|  | *TP53* | c.731delG | p.Gly244AlafsTer3 | 12.17 | Pathogenic |
| Abbreviations: *ARID1A*: AT-rich interaction domain 1A; *ATRX*: ATRX chromatin remodeler; *CCNE1*: cyclin E1; *CDKN1B*: cyclin dependent kinase inhibitor 1B*; ERBB3*: erb-b2 receptor tyrosine kinase 3; *ERBB4*: erb-b2 receptor tyrosine kinase 4; *FANCD2*: FA complementation group D2; *FBXW7*: F-Box And WD Repeat Domain Containing 7; *KRAS*: KRAS proto-oncogene. GTPase; *MSH6*: mutS homolog6; *NOTCH2*: notch receptor 2; *PIK3CA*: phosphatidylinositol-4.5-bisphosphate 3-kinase catalytic subunit alpha; *POLE*: DNA polymerase epsilon*; RB1*: RB transcriptional corepresor 1; *TP53*: tumor protein p53; *RNF43*: ring finger protein; VAF: variant allele frequency; VUS: variant of unknown significance. | | | | | |

| **Table S5**: Comparison of the clinico-pathological parameters of the patients according to the presence of pathogenic variants on their basal plasma samples | | | | | |
| --- | --- | --- | --- | --- | --- |
|  |  | **All patients** | **Basal-alteration** | | **Statistical**  **significance** |
| **Characteristics** | |  | **Non-pathogenic** | **Pathogenic** |  |
| n (%) |  | 26 | 10 (38.5) | 16 (61.5) |  |
| Age (years)* |  | 67.5 (60.1-75.2) | 63.7 (60.4-69.7) | 71.1 (59.7-75.8) | n.s. |
| Sex (n. %) | Male | 14 (53.8) | 7 (70) | 7 (43.8) | n.s. |
|  | Female | 12 (46.2) | 3 (30) | 9 (56.2) |  |
| Treatment at diagnosis (n. %) | Surgery | 4 (15.4) | 2 (20) | 2 (12.5) | n.s. |
|  | NAC | 22 (84.6) | 8 (80) | 14 (87.5) |  |
|  | Disease control-yes | 12 (54.5) | 8 (100) | 4 (28.6) | p < 0.01 |
|  | Disease control-not | 10 (45.5) | 0 | 10 (71.4) |  |
| cT (n. %) | T1 | 3 (11.5) | 3 (30) | 0 | n.s. |
|  | T2 | 9 (34.6) | 4 (40) | 5 (31.2) |  |
|  | T3 | 8 (30.8) | 2 (20) | 6 (37.5) |  |
|  | T4 | 6 (23.1) | 1 (10) | 5 (31.2) |  |
| cN (n. %) | N0 | 13 (50) | 4 (40) | 9 (56.2) | n.s. |
|  | N+ | 13 (50) | 6 (60) | 7 (43.8) |  |
| CA 19-9 (U/ml)* |  | 350.5 (105.8-2203.8) | 71.0 (2.3-305.3) | 1,515.0 (2,69.8-3,418.3) | p< 0.01 |
| Abbreviations: CA. carbohydrate antigen; NA. neoadyuvant treatment; n.s.. no significant. Mann-Whitney U test was employed to compare quantitative variables and Fisher’s exact test for categorical variables. In the non-pathogenic group were including those patients without any alteration in the basal sample. *Quantitative variables are described as median (Q1-Q3). | | | | | |

| **Table S6**: Comparison of the clinico-pathological parameters of the patients according to the presence of KRAS mutations on their basal plasma samples available | | | | | | |
| --- | --- | --- | --- | --- | --- | --- |
|  | |  | **All patients** | **KRAS mutation** | | **Statistical**  **significance** |
| **Characteristics** | |  |  | **No** | **Yes** |  |
| n (%) |  | | 26 | 19 (73.1) | 7 (26.9) |  |
| Age (median. Q1-Q3) |  | | 67.5 (60.1-75.2) | 67.4 (60.4-75.2) | 68.6 (58.9-72.7) | n.s. |
| Sex (n. %) | Male | | 14 (53.8) | 10 (55.6) | 4 (50) | n.s. |
|  | Female | | 12 (46.2) | 8 (44.4) | 4 (50) |  |
| Treatment at diagnosis (n. %) | Surgery | | 4 (15.4) | 3 (16.7) | 1 (12.5) | n.s. |
|  | NAC | | 22 (84.6) | 15 (83.3) | 7 (87.5) |  |
|  | Disease control-yes | | 12 (54.5) | 10 (66.7) | 2 (28.6) | n.s. |
|  | Disease control-not | | 10 (45.5) | 5 (33.3) | 5 (71.4) |  |
| cT (n. %) | T1 | | 3 (11.5) | 3 (16.7) | 0 | n.s. |
|  | T2 | | 9 (34.6) | 6 (33.3) | 3 (37.5) |  |
|  | T3 | | 8 (30.8) | 6 (33.3) | 2 (25) |  |
|  | T4 | | 6 (23.1) | 3 (16.7) | 3 (37.5) |  |
| cN (n. %) | N0 | | 13 (50) | 8 (44.4) | 5 (62.5) | n.s. |
|  | N+ | | 13 (50) | 10 (55.6) | 3 (37.5) |  |
| CA 19-9 (U/ml)* |  | | 350.5 (105.8-2,203.8) | 290.5 (62.5-805.5) | 1,515.0 (247.5-2,352.3) | n.s. |
| Abbreviations: CA. carbohydrate antigen; NAC. neoadyuvant chemotherapy; n.s.. no significant. Mann-Whitney U test was employed to compare quantitative variables and Fisher’s exact test for categorical variables. In the non-pathogenic group were including those patients without any alteration in the basal sample. *Quantitative variables are described as median (Q1-Q3). | | | | | | |
